# Traditional medicine users in a treated chronic disease population: a cross-sectional study in Indonesia

**DOI:** 10.1186/s12906-023-03947-4

**Published:** 2023-04-14

**Authors:** Ivan Surya Pradipta, Kevin Aprilio, Raden Maya Febriyanti, Yozi Fiedya Ningsih, Mochammad Andhika Aji Pratama, Raden Bayu Indradi, Vesara Ardhe Gatera, Sofa Dewi Alfian, Aulia Iskandarsyah, Rizky Abdulah

**Affiliations:** 1 Department of Pharmacology and Clinical Pharmacy, Faculty of Pharmacy, Universitas Padjadjaran, Jalan Ir. Soekarno KM. 21, Jatinangor, Sumedang, West Java 45363 Indonesia; 2 Drug Utilization and Pharmacoepidemiology Research Group, Center of Excellence in Higher Education for Pharmaceutical Care Innovation, Universitas Padjadjaran, Jalan Ir. Soekarno KM. 21, Jatinangor, Sumedang, West Java 45363 Indonesia; 3 Department of Pharmaceutical Biology, Faculty of Pharmacy, Universitas Padjadjaran, Jalan Ir. Soekarno KM. 21, Jatinangor, Sumedang, West Java 45363 Indonesia; 4 Department of Pharmacy and Health Sciences, Universiti Kuala Lumpur - Royal College of Medicine Perak, Ipoh, Perak Malaysia; 5 Department of Clinical Psychology, Faculty of Psychology, Universitas Padjadjaran, Jalan Ir. Soekarno KM. 21, Jatinangor, Sumedang, West Java 45363 Indonesia

**Keywords:** Traditional medicine, Chronic disease, Patient characteristic, Rational drug use

## Abstract

**Background:**

Traditional medicine (TM) is commonly used as a treatment in Indonesia. This raises the need for an analysis of its potential development and irrational use. Therefore, we analyze the proportion of TM users among chronic disease patients and its associated characteristics to optimize the use of TM in Indonesia.

**Methods:**

A cross-sectional study of treated adult chronic disease patients was conducted using the fifth Indonesian Family Life Survey (IFLS-5) database. Descriptive analysis was used to identify the proportion of TM users, while a multivariate logistic regression was used to analyze their characteristics.

**Results:**

This study included 4901 subjects and identified 27.1% as TM users. The highest TM use was in subjects with cancer (43.9%), liver issues (38.3%), cholesterol issues (34.3%), diabetes (33.6%), and stroke (31.7%). Characteristics associated with TM users were a perception of one's current health as unhealthy (OR 2.59, 95% CI 1.76–3.81), low medication adherence (OR 2.49, 95% CI 2.17–2.85), age above 65 years (OR 2.17, 95% CI 1.63–2.90), having higher education (OR 1.64, 95% CI 1.17–2.29), and residence outside of Java (OR 1.27, 95% CI 1.11–1.45).

**Conclusions:**

Low medication adherence among TM users highlights the potentially irrational use of treatment in chronic diseases. Nevertheless, the longstanding use of TM users indicates the potential for its development. Further studies and interventions are needed to optimize TM use in Indonesia.

**Supplementary Information:**

The online version contains supplementary material available at 10.1186/s12906-023-03947-4.

## Introduction

Despite the expansion of modern medicine—and the general improvement in its quality [1]—traditional medicine (TM) is still commonplace in various parts of the world. In 2018, the World Health Organization (WHO) reported that 88% of its 194 member states acknowledge using traditional, complementary, and alternative medicine [2]. While the use of herbal medicine has been increasing in several developed countries, such as the United States [3], its use in developing countries has generally reached a "steady state" where it coexists with modern medicine, even in urban areas [4].

The knowledge and use of TM have been increasing in light of the COVID-19 pandemic [5]. With the pressure the COVID-19 pandemic has imposed on public health—and the increase in TM use—it can be seen that despite the merits traditional herbal medicine offers in terms of treating COVID-19 [6], its use outside the modern medicine system indicates its role as a substitute for quality healthcare within the context of health-seeking behavior [7, 8], mainly due to various issues inherently related to self-medication with herbal medicines [9–11].

The longstanding use of TM has also led to its prominence in societies such as Indonesia [12]. Among the multiplicity of Indonesian traditional medical systems [9], *jamu* is one of the TM generally used in Indonesia [13]. Historically, the term *jamu* stems from Javanese language meaning traditional medicine from plants, minerals, animal parts, or extracts thereof [12]. *Jamu* is now used to generally describe herbal TM of Indonesian origin [12].

TM in the forms of herbal medicine has also been the main concern of Indonesian government [14]. Indonesian government, through its *Badan Pengawas Obat dan Makanan*, has classified herbal medicine into three classifications based its level of evidence: (1) *jamu* as herbal preparations based solely on its empirical evidence; (2) *obat herbal terstandar* (*lit*. standardized herbal medicine) as herbal preparations that have undergone preclinical trials for its safety; and (3) *fitofarmaka* (*lit*. phytomedicine) as herbal preparations that undergone clinical trials for its efficacy [15].

However, despite efforts by the Indonesian government to regulate TM use [16–18], TM is still commonly used freely outside of the formal medical system [13]. Regardless of TM's economic value [19, 20], the lack of scientific evidence in most of TM has prevented its clinical use and integration into the modern medical system, hence posing possibilities for its irrational use [21–24].

Multiple factors contribute to the use of TM, such as perceived illness, demographic and socioeconomic factors, and communication systems [25–27]. Such characteristics correlated with the use of TM can be assumed to arise as the result of social dynamics that influence people's health-seeking behavior. Therefore, the population of TM users can then be used as a proxy to identify groups who are underserved by modern medicine and seek access to traditional herbal medicine as an alternative [28].

A previous study in Indonesia indicated that having a chronic disease is associated with the use of TM [26]. Furthermore, the dependence of people with chronic diseases on long-term treatment, particularly medications, can be assumed to provide a baseline of interpretation regarding the use of TM in society [29]. The length of such diseases should also provide consistent data regarding the use of TM, leading to the interest in studying this phenomenon among chronic disease patients.

Although previous smaller-scale studies have analyzed characteristics of TM users [25–27], the evaluation of such phenomena has been lacking in the context of its relevance to medicine development and its rational use, particularly juxtaposed to the treatment process of chronic disease. With the issues surrounding the current treatment modalities for chronic disease [30], it is essential to identify the real-world conditions of TM users. Therefore, we analyze the proportion and characteristics of TM users in patients with chronic diseases to optimize the use of TM in Indonesia.

## Methods

### Study design and participants

This study was based on the fifth Indonesian Family Life Survey (IFLS-5) database. The IFLS-5 data were obtained from a survey conducted from 2014 to 2015 by RAND Labor and Population in collaboration with Universitas Gadjah Mada [31]. The RAND Corporation, the initiator of IFLS-5, is a policy institute based in Santa Monica, CA, USA [32]. The IFLS-5 survey was performed on approximately 75,000 populations spread over 27 Indonesian provinces, representing approximately 83% of the Indonesian population [33]. The survey was performed using multistage stratified sampling by selecting a random household member from a random household in each enumeration area [33].

In this study, subjects were selected from the IFLS-5 database, with the criterion being chronic disease patients undergoing any type of treatment. Subjects with incomplete data were excluded. Subsequently, subjects were divided into TM users and non-TM users based on observation of the outcome variable as mentioned below.

### Variables and measures

The exposure variables were selected based on the factors of health-seeking behavior that exist in the IFLS-5 database [34]. Relevant variables were divided into two main factors: sociodemographic factors and patient-related factors. Sociodemographic factors include sex, annual income, wealth index, ethnicity, residential area, and insurance ownership. Patient-related factors include education level, smoking behavior, working days missed in a month, current self-perceived health status, and medication adherence.

The outcome variable was defined as treatments used by subjects for their chronic disease. This was obtained on a self-reported basis through the question *"Has a doctor/paramedic/nurse/midwife ever told you that you had [types of chronic disease]?"* (Book IIIB/CD05) [33]. The subsequent question, *"Are you taking [types of medication] to treat [types of chronic disease] and its complications?"* (Book IIIB/CD09a) was posed to each chronic disease subject [33].

Non-TM users were classified as subjects who solely used modern medicine—i.e., pharmacological and nonpharmacological treatments, including radiotherapy, physical/occupational therapy, and psychological therapy. TM users were classified as subjects who reported TM use for their chronic disease, solely or in conjunction with modern medicine. Further details on the operational definition of this study can be found in Table S1.

### Data analysis

A descriptive analysis was performed to describe the proportion of subjects' characteristics, types of chronic disease, and use of traditional or non-TM. A bivariate logistic analysis was used to determine variables potentially used in the multivariate analysis by the cutoff value of *p* < 0.25. The variance inflation factor (VIF) was further analyzed to eliminate multicollinearity. A subsequent multivariate logistic analysis with the enter step was used to analyze the characteristics of TM users. The significance of each exposure variable was determined by a cutoff value of *p* < 0.05 and a 95% confidence interval (95% CI).

All statistical analyses were performed using SPSS® Statistics for Windows™ version 26 from IBM® Corporation (Armonk, NY, USA). The results of this study were then reported following the STROBE (Strengthening the Reporting of Observational Studies in Epidemiology Statement) guidelines for cross-sectional study (Table S2) [35].

## Results

**Fig. 1:**
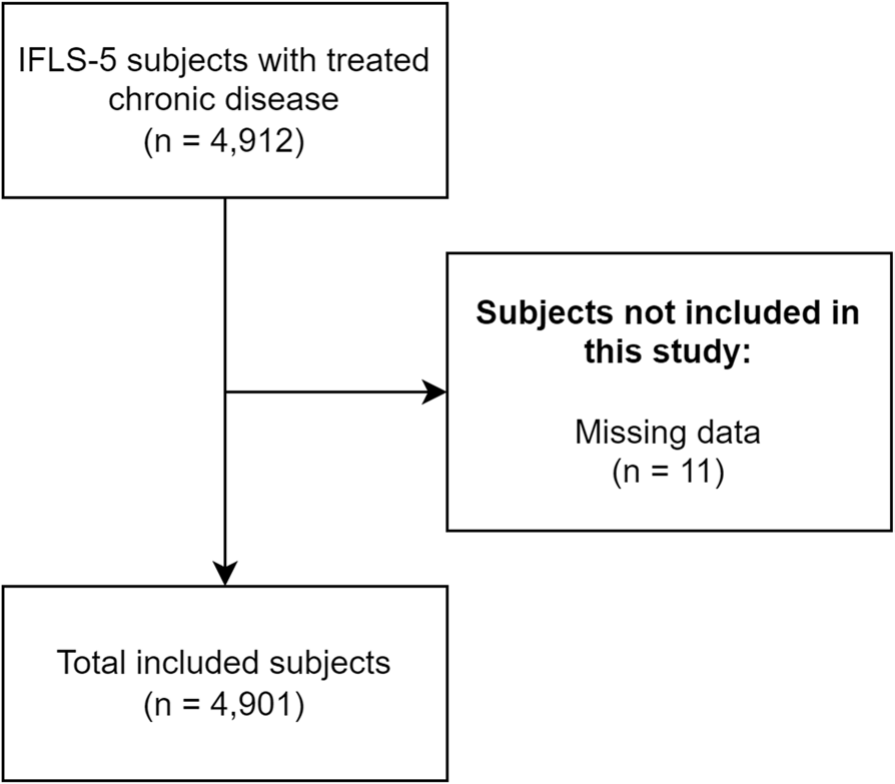
Flow diagram of included subjects

A total of 4,912 subjects from the IFLS-5 database were found to have the chronic disease(s) being treated. Among them, 11 subjects (0.05%) had missing data; 4901 subjects were finally included for further analysis. The flow diagram of the included subjects is presented in Fig. 1.

The predominant characteristics of the population of this study are subjects aged 25–65 years (74.9%), nonsmokers (67.9%), female sex (61.3%), and non-Javanese ethnicity (55%). Subjects who resided in Java dominated the study (58,6%) and most also lived in urban locations (64.6%). As many as 27.1% of subjects were found to use TM. These subjects' characteristics are presented in Table 1.

**Table 1:** Subject characteristics (*n* = 4,901)

No	Characteristics	Proportion (%)
1	Socioeconomic factors	
	Male^a^	38.7%
	Age (years)	
	15–24	8.3%
	25–65	74.9%
	> 66	16.8%
	Annual income (IDR)^a^	
	> 40 million	7.9%
	12–40 million	20.6%
	< 12 million	32.4%
	Not working	38.3%
	Wealth index^a^	
	Quintile 5	24.5%
	Quintile 4	18.9%
	Quintile 3	22.4%
	Quintile 2	18.9%
	Quintile 1	14.9%
	Non-Javanese ethnicity^a^	55.0%
	Non-Java residence^a^	41.4%
	Rural residence^a^	35.4%
	No insurance ownership^a^	44.3%
2	Patient-related factors	
	Education	
	Unschooled	8.0%
	Elementary	35.9%
	Junior high	16.1%
	Senior high	25.4%
	Higher education	13.9%
	Smoking behavior	
	Non-smoker	67.9%
	Ex-smoker	10.1%
	Active smoker	22.0%
	Active days missed in a month^a^	
	0	37.3%
	1–7	47.0%
	> 7	15.6%
	Current self-related health status	
	Very healthy	8.6%
	Somewhat healthy	45.8%
	Somewhat unhealthy	40.7%
	Very unhealthy	4.8%
	Took no medications in the past week	44.3%
3	Traditional medicine users	27.1%

^a^missing value: Sex 1 (0.0%), Education 36 (0.7%), Annual income 38 (0.8%), Wealth index 22 (0.4%), Ethnicity 22 (0.4%), Geographical residence 1 (0.0%), Demographical residence 1 (0.0%), Insurance ownership 20 (0.4%), Active days missed in a month 4 (0.1%)

**Fig. 2:**
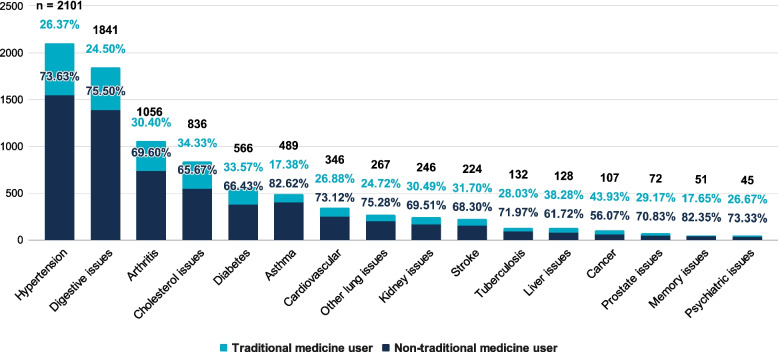
Distribution of treatments across chronic diseases

Chronic diseases such as cancer (43.9%), liver issues (38.3%), cholesterol issues (34.3%), diabetes (33.6%), and stroke (31.7%) had the highest number of TM users by proportion. This contrasts with chronic diseases such as memory issues (17.6%), digestive issues (24.5%), other lung issues (24.7%), hypertension (26.4%), and psychiatric issues (26.7%), which had the lowest number of TM users by proportion. The proportion of TM users in each chronic disease is presented in Fig. 2.

In the multivariate analysis, all variables used in this analysis exhibited no multicollinearity by having a value of VIF < 10 (Table S3). It was identified that subjects with poor health perception (OR 2.59, 95% CI 1.76–3.81), low medication adherence (OR 2.49, 95% CI 2.17–2.85), > 65 years of age (OR 2.17, 95% CI 1.63–2.90), having higher education (OR 1.64, 95% CI 1.17–2.29), and residence outside of Java (OR 1.27, 95% CI 1.11–1.45) were more likely to use TM. A lower perception of one's health, poor medication adherence, and older age were the most significant variables contributing to the use of TM. The multivariate analysis is presented in Table 2.

**Table 2:** Characteristics of traditional medicine users (*n* = 4,901)

No	Variables	Non-traditional medicine users (*n* = 3,573)	Traditional medicine users (*n* = 1,328)	Bivariate		Multivariate	
				**Odds ratio (95% CI)**	***p*-value**	**Odds ratio (95% CI)**	***p*-value**
1	Socioeconomic factors						
	Male	1324	572	1.29 (1.13–1.46)	0.000*	1.08 (0.88–1.31)	0.462
	Age (years)						
	15–24	336	71	Reference			
	25–65	2615	1055	1.54 (1.14–2.08)	0.005*	1.82 (1.30–2.56)	0.001**
	> 65	622	202	1.91 (1.46–2.49)	0.000*	2.17 (1.63–2.90)	0.000**
	Annual income (IDR)						
	> 40 million	262	126	Reference			
	12–40 million	714	296	0.68 (0.54–0.87)	0.002*	0.96 (0.72–1.27)	0.751
	< 12 million	1157	432	0.78 (0.61–0.99)	0.038*	0.93 (0.70–1.23)	0.612
	No income	1413	463	0.86 (0.67–1.11)	0.248*	0.91 (0.69–1.20)	0.481
	Wealth index						
	Quintile 5	866	334	Reference			
	Quintile 4	663	263	1.03 (0.85–1.25)	0.772	1.05 (0.86–1.28)	0.624
	Quintile 3	797	303	0.99 (0.82–1.19)	0.878	0.99 (0.82–1.20)	0.930
	Quintile 2	673	253	0.98 (0.81–1.18)	0.794	1.01 (0.82–1.24)	0.924
	Quintile 1	557	170	0.79 (0.64–0.98)	0.031*	0.83 (0.66–1.04)	0.103
	Non-Javanese ethnicity	1957	737	1.03 (0.91–1.17)	0.674	Not Included	
	Non-Java residence	1414	616	1.32 (1.16–1.50)	0.000*	1.27 (1.11–1.45)	0.001**
	Rural residence	1247	489	1.08 (0.95–1.24)	0.214*	1.03 (0.89–1.20)	0.663
	No insurance ownership	1565	607	1.09 (0.96–1.23)	0.204*	1.10 (0.96–1.26)	0.183
2	Patient-related factors						
	Education						
	Unschooled	301	89	Reference			
	Elementary	1294	465	1.22 (0.94–1.58)	0.140*	1.15 (0.87–1.51)	0.337
	Junior high	585	203	1.17 (0.88–1.56)	0.272	1.12 (0.82–1.54)	0.478
	Senior high	900	346	1.30 (1.00–1.70)	0.054*	1.25 (0.92–1.69)	0.160
	Higher education	464	218	1.59 (1.19–2.12)	0.002*	1.64 (1.17–2.29)	0.004**
	Smoking behavior						
	Non-smoker	2490	839	Reference			
	Ex-smoker	346	149	1.28 (1.04–1.57)	0.020*	1.25 (0.96–1.62)	0.093
	Active smoker	737	340	1.37 (1.18–1.60)	0.000*	1.15 (0.93–1.43)	0.198
	Active days missed in a month						
	0	1330	497	Reference		Not Included	
	1–7	1690	614	0.97 (0.85–1.12)	0.690		
	> 7	550	216	1.05 (0.87–1.27)	0.605		
	Current self-rated health status						
	Very healthy	341	81	Reference			
	Somewhat healthy	1635	612	1.58 (1.22–2.04)	0.001*	1.54 (1.18–2.02)	0.002**
	Somewhat unhealthy	1440	557	1.63 (1.25–2.12)	0.000*	1.87 (1.42–2.45)	0.000**
	Very unhealthy	157	78	2.09 (1.45–3.01)	0.000*	2.59 (1.76–3.82)	0.000**
	Took no prescribed medications in the past week	1375	793	2.37 (2.08–2.70)	0.000*	2.49 (2.17–2.85)	0.000**

The multivariate model was validated by Hosmer–Lemeshow test (χ^2^ = 6.960 and *p* = 0.541) and omnibus test (χ^2^ = 285.299 and *p* = 0.000). By 2014–2015, 1 USD is averagely equivalent to 13,118 IDR

^*^Univariate analysis statistically significant by *p* < 0.25

^**^Multivariate analysis statistically significant by *p* < 0.05

## Discussion

This study found that older age, non-Java residence, higher education, poor self-perceived health status, and nonadherence to prescribed medication are associated with TM use. These findings highlight that knowledge (education level and age), access (geographical residence), and perception of health (self-perceived health status and medication adherence) are the three main factors associated with the use of TM. The characteristics found in this study may explain the social phenomena and conditions surrounding chronic disease treatment in Indonesia. It can be a guide to identify potential problems and strategies to optimize the benefits of TM use.

### Knowledge

In our study, knowledge was associated with the utilization of TM. It refers to the knowledge of their illness and the treatment available. This concept is in line with the health-seeking behavior theory that the pursuit of treatment is largely driven by perception and knowledge of illness [8, 36].

A previous study showed that the association between increasing use of TM and education levels indicates the correlation between health knowledge and the options of treatment modalities for such diseases [37]. As TM in Indonesia is used empirically, information regarding it is disseminated through daily social functions [37]. Harahap et al. also indicated a similar finding in the form of higher self-medication rates among patients with higher education [38].

The association of TM use with increased age should also be seen as an indicator of one's knowledge of TM. The increasing need for healthcare with age leads to an increase in exposure to medicine—including TM—and therefore increases one's knowledge of the traditional means of treatment available for the condition [37]. This process is also in line with the general tendency of chronic diseases to have their onset in later stages of one's life.

While the use of TM may indicate patients' better knowledge, the self-obtained nature of TM knowledge may pose problems [39]. The lack of assessment from a healthcare professional may lead to phenomena such as healer shopping [40], causing issues related to treatment fragmentation [41]. In this regard, the use of TM poses potential treatment irrationality through the possible intervention of the treatment process at large as well as improper use of the TM itself.

### Access

Despite efforts by the Indonesian government to overcome the disparity between Indonesian regions, the problem of economic and development disparity can still be seen [42]. In healthcare, this problem presents itself in various forms, such as the lower rate of utilization of outpatient care facilities in rural areas [43]. While this study does not show a discrepancy in TM use across demographical residences, the difference in TM use between Java and other Indonesian areas should indicate problems in access to health facilities and modern medicine due to geographical development disparity [44].

In such a sense, the persisting use of TM may further indicate a larger issue regarding the formal medical system itself, as signified by Alkaff et al. [30]. Therefore, it can be assumed that issues regarding healthcare access in the Indonesian context are related to the medical system itself, particularly owing to the increasing public healthcare coverage in Indonesia by BPJS Kesehatan [45]. The phenomenon may also signify other factors influencing the use of TM, i.e., cultural factors.

### Health perception

A study by Widayanti et al. concluded that, within the context of multiple medical systems in Indonesia [46], the perception of the medical system was among the main factors influencing the treatment of choice [9]. This aspect is most apparent in the association of TM use with treatment nonadherence, as one of the fundamental issues in treatment irrationality. Additionally, the role of perception also emerged from the association of low self-perceived health status to TM use without the impairment of a health condition (as measured in working days missed in a month). This shows the significance of subjectivity and perception of sickness within the context of health-seeking behavior [8].

A study by Iskandasryah et al. revealed that TM use was associated with a lower perception of one's health condition and eventually with patients' treatment adherence [47], confirming the association of lower adherence to TM use. However, it should also be noted that patients' difficulties in accessing modern medicine, which often causes which causes its substitution with the more easily accessible TM [7], may also lead to treatment nonadherence.

The role TM plays in building health perception raises its potential for development. While being commonly used as a substitution for modern medicine [48], the empirical basis of various TM use—for instance, *jamu*—is mostly yet to be clinically confirmed as a relatively safe and effective means of treatment [49]. Therefore, the acceptance of *jamu* in the formal medical system through scientific research and evidence should be developed to widen access to TM in treatment and reduce its potential irrational use.

## Study strengths and limitations

The self-reported data in this study might pose recall and social-desirability biases. Furthermore, the inferential nature of this study, using secondary data obtained through structured questionnaires, may pose a reduction of perceived phenomena that would require further study through qualitative means. Due to the limited data in the database, this study did not consider variables that may affect the study outcome, such as severity staging, length of the disease, and characterization of the traditional medical system types and the forms of medicine used. In the study design aspect, the causality between exposure and outcome variables should be interpreted cautiously since time differences between the variables cannot be considered in the cross-sectional study. However, the national scope of IFLS-5 might make the results of this study applicable to the general Indonesian population. Acceptable missing data can contribute to internal and external validities. Furthermore, we followed international consensus reporting guidelines to provide a systematic and transparent report.

## Recommendations

**Fig. 3:**
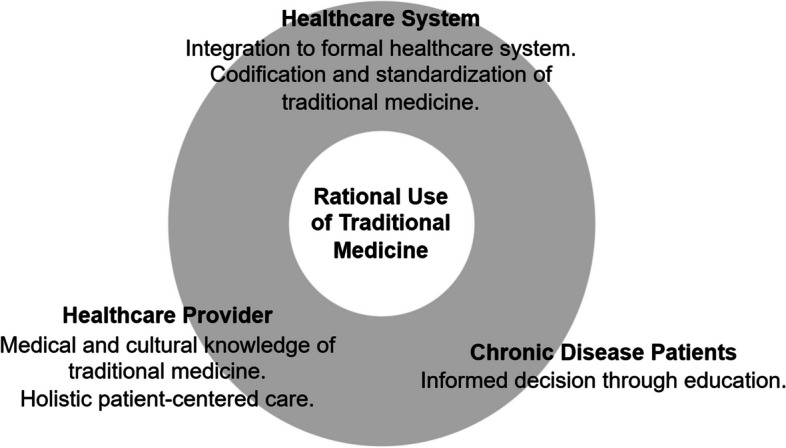
Recommendations for improving rational use of traditional medicine

Our study highlights that TM use among chronic diseases can be seen from two sides. First, it can exacerbate the potential irrational use of medicine due to nonadherence, lack of scientific evidence, and unregulated use. Second, TM can be potentially developed as an accessible alternative treatment for chronic disease patients. Therefore, several recommendations to optimize TM use can be considered that involve three essential aspects, i.e., patient, healthcare provider, and healthcare system (Fig. 3).

Firstly, educating patients with chronic diseases about the disease and TM should be optimized. It requires medical practitioners to have a more patient-centered approach by addressing patients' issues and concerns regarding their chronic diseases and TM use related to those concerns. Furthermore, it also requires further improvements in access to quality healthcare to allow healthcare professionals to monitor TM use and prevent its potential irrationalities.

Secondly, medical practitioners should have the cultural knowledge to identify TM commonly used in chronic diseases and its efficacy, safety, and interaction. Further cultural sensitivity is also needed in addressing issues that might emerge with patients' concerns.

Thirdly, the system and practice of TM should be formalized to the extent possible while preserving its cultural values. Further research and standardization on TM should be performed to provide a consistent framework, ensure safety and efficacy, and enable patients to make well-informed decisions about their treatment. Providing scientific evidence can also increase the acceptance and use of TM in the formal medical system, allowing the integration of TM use into the formal healthcare system.

## Conclusions

TM is still widely used for the treatment of various chronic diseases in Indonesia, indicating its potential for further development. However, nonadherence and uncontrolled TM use indicate potential issues of its irrational use. Therefore, further development of strategies to optimize TM use in Indonesia is needed.

### Supplementary Information

**Additional file 1:** **Table S1.** Operational definition of study. **Table S2.** STROBE statement checklist for cross-sectional study. **Table S3.** Variance Inflation Factor (VIF) of variables.  

## Data Availability

The IFLS-5 dataset used in this study is available at https://www.rand.org/well-being/social-and-behavioral-policy/data/FLS/IFLS/access.html.
