# Correction: Traditional medicine users in a treated chronic disease population: a cross-sectional study in Indonesia

**DOI:** 10.1186/s12906-023-04276-2

**Published:** 2023-12-06

**Authors:** Ivan Surya Pradipta, Kevin Aprilio, Raden Maya Febriyanti, Yozi Fiedya Ningsih, Mochammad Andhika Aji Pratama, Raden Bayu Indradi, Vesara Ardhe Gatera, Sofa Dewi Alfian, Auliya Iskandarsyah, Rizky Abdulah

**Affiliations:** 1 Department of Pharmacology and Clinical Pharmacy, Faculty of Pharmacy, Universitas Padjadjaran, Jalan Ir. Soekarno KM. 21, Jatinangor, Sumedang, West Java 45363 Indonesia; 2 Drug Utilization and Pharmacoepidemiology Research Group, Center of Excellence in Higher Education for Pharmaceutical Care Innovation, Universitas Padjadjaran, Jalan Ir. Soekarno KM. 21, Jatinangor, Sumedang, West Java 45363 Indonesia; 3 Department of Pharmaceutical Biology, Faculty of Pharmacy, Universitas Padjadjaran, Jalan Ir. Soekarno KM. 21, Jatinangor, Sumedang, West Java 45363 Indonesia; 4 Department of Pharmacy and Health Sciences, Universiti Kuala Lumpur - Royal College of Medicine Perak, Ipoh, Perak Malaysia; 5 Department of Clinical Psychology, Faculty of Psychology, Universitas Padjadjaran, Jalan Ir. Soekarno KM. 21, Jatinangor, Sumedang, West Java 45363 Indonesia

**Correction: BMC Complement Med Ther 23, 120 (2023)**

**https://doi.org/10.1186/s12906-023-03947-4**

We have noticed several errors in the data presentation of our article [1]. These errors do not impact the Results and Discussion section in the article, as the interpretation was made based on the correct information. However, we believe these errors might confuse readers’ interpretation of the study. Hence, we see the importance in acknowledging and correcting these errors. We extend our sincere apology for any issues caused by these errors.

Corrections:

In Table 1, the percentage of male subjects in the study should read “38.7%” instead of “8.7%”. The correct number of male subjects representing this percentage has been shown in Table 2 of the original article. The data in Table 1 are otherwise correct.

In Fig. 2, the legend for non-traditional medicine user and traditional medicine user was mixed up. Non-traditional medicine users should have been represented by the dark blue color, while traditional medicine users should have been represented by the light blue color. The proportion of which, presented in the figure, is otherwise correct. Kindly find the corrected Fig. 2 in this erratum.

**Fig. 2:**
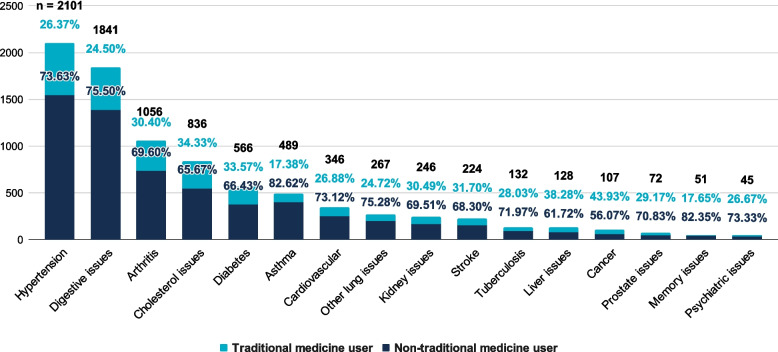
Distribution of treatments across chronic diseases

The original article has been corrected.
