# Supplementary material for: Traditional medicine users in a treated chronic disease population: a cross-sectional study in Indonesia
Source: BMC Complement Med Ther. 2023 Apr 14;23:120. doi: 10.1186/s12906-023-03947-4 (PMC10102674; doi:10.1186/s12906-023-03947-4)
Supplement: Supplementary file 1 — Additional file 1: Table S1. Operational definition of study. Table S2. STROBE statement checklist for cross-sectional study. Table S3. Variance Inflation Factor (VIF) of variables. [file 12906_2023_3947_MOESM1_ESM.pdf]

Supplementary Materials

Title:

Traditional medicine users in a treated chronic disease population: a cross-sectional study in Indonesia

Authors:

Ivan Surya Pradipta, Kevin Aprilio, Raden Maya Febriyanti, Yozi Fiedya Ningsih, Mochammad Andhika Aji Pratama, Raden Bayu Indradi, Vesara Ardhe Gatera, Sofa Dewi Alfian, Auliya Iskandarsyah, Rizky Abdulah

Table S1. Operational definition of study

No.	Variables	Operational Definition
**Exposure**		
1.	Sex	Biological characteristic of subject. This variable was classified into male and female.
2.	Age	Age of subject during the IFLS-5 study. In this study, this variable was classified into < 15 years, 15-24 years, 25-65 years, and > 65 years.
3.	Education	The latest formal education level attended by the subject during the IFLS-5 study. This variable was classified into unschooled; elementary (sekolah dasar/SD); junior high (sekolah menengah pertama/SMP); senior high (sekolah menengah atas/SMA); and higher education, according to the Indonesian education system.
4.	Annual income	Total annual earnings of each subject in Indonesian Rupiah (IDR). In this study, this variable was classified into < 12 million; 12-40 million; and > 40 million.
5.	Wealth index	Classification of subject's household wealth based on United Nations World Food Program principal component analysis (PCA). In this study, this variable was classified into quintiles from the entire IFLS-5 subject population.
6.	Ethnicity	Subject's self-identified ethnicity identity. In this study, this variable was classified into Javanese and non-Javanese.

7.	Geographical residence	Geographical location of the subject's residence during the IFLS-5 study. This variable was measured by the subject's provincial location of residence and classified in this study into Java and non-Java residence.
8.	Demographical residence	Demographical condition of the subject's residence during the IFLS-5 study. This variable was classified into rural and urban residence.
9.	Insurance ownership	Subject's participation or ownership of public and/or private insurance program/policy.
10.	Self-perceived health status	Subject's observation of their health during the IFLS-5 study. This variable was measured with the question *"In general, how is your health?"*, with responses being very healthy; somewhat healthy; somewhat unhealthy; and very unhealthy.
11.	Active days missed in a month	The number of days in the last month of the IFLS-5 study in which subject misses their primary daily activities due to poor health. This variable was measured with the question *"During the last 4 weeks, how many days of your primary daily activities did you miss due to poor health?"*, with responses classified in this study into 0; 1-7; and > 7 days.
12.	Medication adherence	Prescribed medications taken routinely by the subject for each of their chronic diseases. This variable was measured with the question *"In order to deal with [types of chronic disease] are you currently taking prescribed medication on a weekly basis?*", with the response interpreted in this study as subject's adherence to medication.
13.	Smoking behavior	Tobacco consumption behavior of subject since and during the IFLS-5 study. This variable was measured with two questions: *"Have you ever chewed tobacco, smoked a pipe, smoked self-rolled cigarettes, or smoked cigarettes/cigars?"* and "*Do you still have the habit or have you totally quit?"*, with responses classified in this study into non-smoker; ex-smoker; and active smoker.
14.	Types of chronic diseases	Chronic disease being had by the subject during the IFLS-5 study. These chronic diseases were classified by the IFLS-5 study to include arthritis, diabetes, digestive issues, kidney issues, cholesterol issues (by

		total or LDL cholesterol), memory issues, prostate issues, psychiatric issues, asthma, hypertension, cancer (as a separate classification from other diseases), liver issues, other lung issues, stroke, tuberculosis, and cardiovascular issues. This variable was measured with the question *"Has a doctor/paramedic/nurse/midwife ever told you that you had [types of chronic disease]?"* for each chronic disease.
**Outcome**		
15.	Treatment use	Means of treatment used for each chronic disease. This variable was measured with a question *"Are you taking [types of treatment] treatments to treat [types of chronic disease] and its complications?"*, with responses being traditional medicine; modern medicine, including nonpharmacological therapies such as radiotherapy, physical/occupational therapy, and psychological treatment; and other treatment. This variable was classified in this study into traditional medicine user and nontraditional medicine user, based on the subject's traditional medicine use.

Table S2. STROBE statement checklist for cross-sectional study

**Section**	**Item no.**	**Recommendation**	**Page no.**
**Title and abstract**			
	1.	(a) Indicate the study's design with a commonly used term in the title or the abstract	1
		(b) Provide in the abstract an informative and balanced summary of what was done and what was found	1
**Introduction**			
Background or rationale	2.	Explain the scientific background and rationale for the investigation being reported	2
Objectives	3.	State specific objectives, including any prespecified hypotheses	2

**Methods**			
Study design	4.	Present key elements of study design early in the paper	2-3
Setting	5.	Describe the setting, locations, and relevant dates, including periods of recruitment, exposure, follow-up, and data collection.	2
Participants	6.	Give the eligibility criteria, and the sources and methods of selection for participants	2
Variables	7.	Clearly define all outcomes, exposures, predictors, potential confounders, and effect modifiers. Give diagnostic criteria, if applicable.	2-3<br>Table S1*
Data sources or measurement	8.	For each variable of interest, give sources of data and details of methods of assessment (measurement). Describe comparability of assessment methods of there is more than one group.	Table S1*
Bias	9.	Describe any efforts to address potential sources of bias	N/A
Study size	10.	Explain how the study size was arrived at	3<br>Figure 1
Quantitative variables	11.	Explain how quantitative variables were handled in the analyses. If applicable, describe which groupings were chosen and why	Table S1*
Statistical methods	12.	(a) Describe all statistical methods, including those used to control for confounding	3
		(b) Describe any methods used to examine subgroups and interactions	N/A
		(c) Explain how missing data were addressed	2<br>Figure 1
		(d) If applicable, describe analytical methods taking account of sampling strategy	N/A
		(e) Describe any sensitivity analyses	N/A
**Results**			

Participants	13.	(a) Report numbers of individuals at each stage of study—eg numbers potentially eligible, examined for eligibility, confirmed eligible, included in the study, completing follow-up, and analysed	3<br>Figure 1
		(b) Give reasons for non-participation at each stage	3<br>Figure 1
		(c) Consider use of a flow diagram	3<br>Figure 1
Descriptive data	14.	(a) Give characteristics of study participants (eg demographic, clinical, social) and information on exposures and potential confounders	3-4<br>Table 1<br>Figure 2
		(b) Indicate number of participants with missing data for each variable of interest	4<br>Table 1
Outcome data	15.	Report numbers of outcome events or summary measures	3-4<br>Table 1
Main results	16.	(a) Give unadjusted estimates and, if applicable, confounder-adjusted estimates and their precision (eg, 95% confidence interval). Make clear which confounders were adjusted for and why they were included.	3, 6<br>Table 2<br>Table S3*
		(b) Report category boundaries when continuous variables were categorized	Table S1*
		(c) If relevant, consider translating estimates of relative risk into absolute risk for a meaningful time period	N/A
Other analyses	17.	Report other analyses done—eg analyses of subgroup interactions, and sensitivity analyses	N/A
**Discussion**			
Key results	18.	Summarize key results with reference to study objectives	4

Limitation	19.	Discuss limitations of the study, taking into account sources of potential bias or imprecision. Discuss both direction and magnitude of any potential bias	5
	20.	Give a cautious overall interpretation of results considering objectives, limitations, multiplicity of analyses, results from similar studies, and other relevant evidence	4-5
Generalizability	21.	Discuss the generalizability (external validity) of the study results	2, 5
Other information			
Funding	22.	Give the source of funding and the role of the funders for the present study and, if applicable, for the original study on which the present article is based.	7

Note: * = sources from Supplementary Materials; N/A = not applicable

Table S3. Variance Inflation Factor (VIF) of variables

**Exposure Variable**	**VIF**
Sex	1.968
Age	1.178
Education	1.433
Annual income	1.251
Wealth index	1.072
Ethnicity	1.232
Geographical residence	1.272
Demographical residence	1.149
Insurance ownership	1.072
Self-perceived health status	1.247
Active days missed in a month	1.236
Medication adherence	1.050
Smoking behavior	1.885
